# The analysis of neutron reflectivity from Langmuir monolayers of lipids using molecular dynamics simulations: the role of lipid area

**DOI:** 10.1098/rsos.241727

**Published:** 2025-03-19

**Authors:** Arwel V. Hughes, Valeria Losasso, Martyn Winn

**Affiliations:** ^1^ ISIS Pulsed Neutron and Muon Source, Science and Technology Facilities Council, Rutherford Appleton Laboratory, Didcot, Oxfordshire OX11 OQX, UK; ^2^ Science and Technology Facilities Council, Daresbury Laboratory, Warrington WA4 4AD, UK; ^3^ Science and Technology Facilities Council, Research Complex at Harwell, Rutherford Appleton Laboratory, Didcot OX11 0FA, UK

**Keywords:** neutron reflectivity, lipid layers, molecular dynamics

## Abstract

Biomolecular simulations are increasingly being used to generate detailed structural models to aid interpretation of neutron reflectometry (NR) data obtained from model biological membranes. Unlike globular systems, often studied by small-angle scattering, simulations of two-dimensional layers are sensitive to the simulation cell used which constrains the system laterally. We perform a careful analysis of NR data obtained from a monolayer of the lipid distearoylphosphatidylcholine at the air–water interface and show that the fit of number density profiles obtained from atomistic molecular dynamics simulation to the experimental data is very sensitive to the assumed area per lipid (APL). We propose a protocol for obtaining a realistic isotherm by combining the experimental surface pressure corresponding to a reflectometry measurement with an APL obtained from the simulation that best fits that data. Finally, we demonstrate how downstream interpretation of the experimental sample, derived from structural and dynamic properties of the atomistic model, depends strongly on the correct choice of simulation cell.

## 1. Introduction

Neutron reflectometry (NR) provides detailed structural information on layered materials by combining the deep penetration of neutrons with the ability to achieve high resolution along an axis perpendicular to the layers. For this reason, it is being increasingly applied to pseudo-two-dimensional biological membrane systems, which are difficult to study by other structural methods [1,2]. Improvements in sample preparation have led to ever more complex systems being measured such as multi-component multi-layer model systems [3,4]. Nevertheless, simpler models such as Langmuir–Pockels monolayers have great utility where a full model of a membrane is not required. Consisting of single molecular layers of amphiphiles spread at the air–water interface, they have real utility due to their simplicity, and the fact that complete control over the physical and chemical environment is obtained. Model monolayers have been used for a wide variety of purposes [5], including the structural description of the air–water interface [6]; the modelling of biological processes at the cell membrane surface such as peptide assembly [7] or the interaction between antibiotics and bacterial membranes [8]; the study of self-assembly at the air/liquid interface of surfactants [9] or gold nanoparticles [10]; or as templates for the growth of inorganic crystals [11].

In addition to being penetrating and non-destructive, neutrons can take advantage of isotopic labelling of components with deuterium, coupled with ‘contrast matching’ of the bulk liquid phases (i.e. D_2_O/H_2_O mixing ratio), due to the very different scattering cross-sections of hydrogen and deuterium. This allows individual components of complex mixtures of lipids and proteins to be highlighted, and so NR has been used extensively in the characterization of biologically relevant bilayers and monolayers [12–14]. Traditional methods of interpreting NR data from such systems have been crude, involving subdividing the interface into layers that approximately represent the density variation across the interface, and then minimizing the parameters of those layers (e.g. thickness or density) to optimize agreement between the model and the data [15]. These models have the disadvantage of only allowing very low resolution and simplistic interpretations of the structures of these systems, and there has therefore been a long interest in developing models which extend the interpretation of scattering data by parametrizing the interface in ways based more closely on the chemical structures of the components involved, rather than being tied to approximate layers. An example of this approach was to describe monolayers and bilayers as collections of Gaussians which describe the distributions of ‘submolecular fragments’ with a more conventional biophysical meaning—such as methyl or choline groups for example [16]. After all, the scattering length density (SLD) of a region of space is a function solely of the number and type of atoms per unit volume found there, and where the volume is independently known [17], the SLD can be reasonably constructed from continuous functions representing the spatial distribution of these groups, and the expected scattering profiles calculated from there.

At the time when these distribution models were developed, constructing representative functional distributions was the best that could be practically done, since even though atomistic simulation was of course available, it was prohibitively expensive (in terms of the computational resources required and the time taken for simulations) for routine use. In recent years, however, developments in available computing power (in both software and hardware) mean that the scale of the calculation effort required is no longer prohibitive, with large simulations of complex interfaces becoming feasible on routine timescales, and there has therefore been much recent interest in the use of molecular dynamics (MD) simulations to generate more detailed models for the analysis of scattering data [3,18–20]. Molecular simulation provides *in silico* atomistic-level information on a wide variety of systems, and membrane systems have been widely studied, for instance, to investigate the effects of a lipid environment on protein structures that have been determined by structural biology techniques [21]. However, atomistic molecular simulation is still limited by the timescales and length scales that can be achieved, despite the development of several enhanced sampling schemes [22–26]. Coarse-graining approaches [27–29] overcome these limitations to some extent, but at the cost of losing atomistic detail which may be crucial for lipid–peptide interactions, or for neutrons, detailed information about the interface between the membrane and the bulk phases which—depending on deuteration—may dominate the scattering in certain cases.

Typically, in using MD trajectories to analyse NR data, the comparison is done ‘after the fact’, in that the results of MD simulations are compared with fits of the data from layer models, and conclusions regarding the experimental system inferred [20]. More recently, there has been interest in using structures taken from MD trajectories directly to create models that are then used to fit the experimental data directly [3,18–20]. Clearly, atomistic MD carried out in a small simulation cell does not capture the same ensemble as observed in experiment, most obviously because experimental samples may cover several cm^2^, whereas a simulation box will be a few hundred nm^2^ at most. Additionally, contributions from other aspects of the experimental system that are not modelled in the simulation (such as substrate supports) must be added to the model to describe the actual data signal [18]. Nevertheless, correspondence (or lack of) between simulation and experiment allows the interpretation of the experimental system to benefit from the atomistic resolution of the MD, even though the experimental resolution of the neutron data does not strictly extend this far. The simulation effectively allows the inclusion of prior knowledge, in the form of known chemistry that is encoded in the force field, and additionally where the correspondence between theory and data is strong, the great advantage of the use of MD lies in the possibility of subsequent deeper interpretation of the properties of the interface from an analysis of the MD trajectories and structures. However, for such comparisons to yield meaningful insights, it is necessary that the simulation is of a system that is as close as possible to the state of the measured sample, or erroneous conclusions could be drawn.

Where the correspondence between simulation and experimental data is initially not strong, a common approach is to then use the experimental data to inform the generation of refined MD trajectories to improve the correspondence between sample and simulation, in that restraints derived from experiments can be used to steer the MD towards an ensemble that better reflects the experimental case. This general approach has been applied to a variety of experimental techniques [2], including nuclear magnetic resonance spectroscopy [30,31], cryo-electron microscopy [32], X-ray crystallography [33], electrophysiology [34] or small-angle neutron or X-ray scattering [35]. However, to our knowledge, this approach has not yet been applied to reflectivity data. In this current study, we adopt this approach of using the fit to NR data to guide the choice of simulation cell parameters and hence the ensemble explored by MD. Starting with a range of estimates of the restraints, we use corroboration with experiment to determine the optimal correlation between simulation and data. Here, we study this approach for the monolayer case, where the major experimental variable—and hence the cell parameter restraint in the simulation—is the degree of lateral compression of the monolayer. The key quantity, both experimentally and *in silico*, is the area per lipid (APL).

As a test case, we consider a Langmuir monolayer of the lipid distearoylphosphatidylcholine (DSPC) at the air–water interface. DSPC is a fully saturated long-tail (C18) phospholipid. It occurs naturally in biomembranes and is also a common component in synthetic particles, for example lipid nanoparticles used in the delivery of mRNA vaccines [36] and microbubbles used for ultrasound imaging [37]. More pertinently for this study, it forms stable, easily manipulated monolayers of a clearly defined phase and is available commercially in a variety of deuterated forms, and for which an existing comprehensive dataset already exists [38]. McCluskey *et al.* [19] have previously compared molecular simulations with NR data for DSPC monolayers at a series of compressions, using an all-atom force field, a united atom force field and a coarse-grained force field. In contrast to the current work, the APL fixed in the simulations was taken directly from an experimental isotherm. Here we show that the correct choice of APL is crucial to the analysis, and instead of being set from the experimental isotherm, it is necessary to guide the simulation to the correct unit cell from the reflectivity measurement itself. In total, we consider three variants of the isotherm from a single dataset; the experimentally measured curve, that derived directly from the simulation trajectory, and that obtained from optimizing the simulation to the neutron data. These isotherms are not coincident, and the discrepancies between them are discussed in the context of a structural interpretation of reflectivity measurements, and how erroneous physical conclusions could be drawn by not paying sufficient attention to matching simulation and experiment when analysing data from these systems.

## 2. Methods

### 2.1. Overall workflow

In a Langmuir monolayer experiment, the main experimental variable is the degree of compression of the monolayer, and this is varied continuously by means of movable barriers which physically restrict the available area of the interface. The main observable during the experiment is the surface pressure, which is defined as the difference between the surface tension of the clean and monolayer-covered surface. The pressure is measured continuously during compression, leading to isotherms of component area versus surface pressure, which are highly diagnostic of the monolayer state. Over the course of the isotherm, depending on the monolayer composition and phase, the APL can vary over many decades reflecting changes in monolayer structure. Any attempt to model a monolayer experiment atomistically must therefore account for this area variability.

Although the experimental isotherm is known, the true test of correspondence between theory and experiment is the goodness of fit $\chi^{2}$ of the model (including the atomistically modelled monolayer and any additional layers) to the experimental data. We start from a point where we assume that the APL inferred from the experimental isotherm may not be a true reflection of the monolayer state. Therefore, we carry out simulations at multiple confined areas and compare each with the data, monitoring the results in terms of $\chi^{2}$. Then, from these comparisons, we converge towards the best available model by refining the monolayer confinement to find the best fit. By repeating the process at each lateral pressure, an isotherm can be built up relating the applied lateral pressure to the best fit APL. The final output is a set of atomistically detailed models which best represent the experiment, and which are the basis for downstream interpretation. We now describe these steps in more detail.

### 2.2. Neutron reflectivity

**Figure 1.**
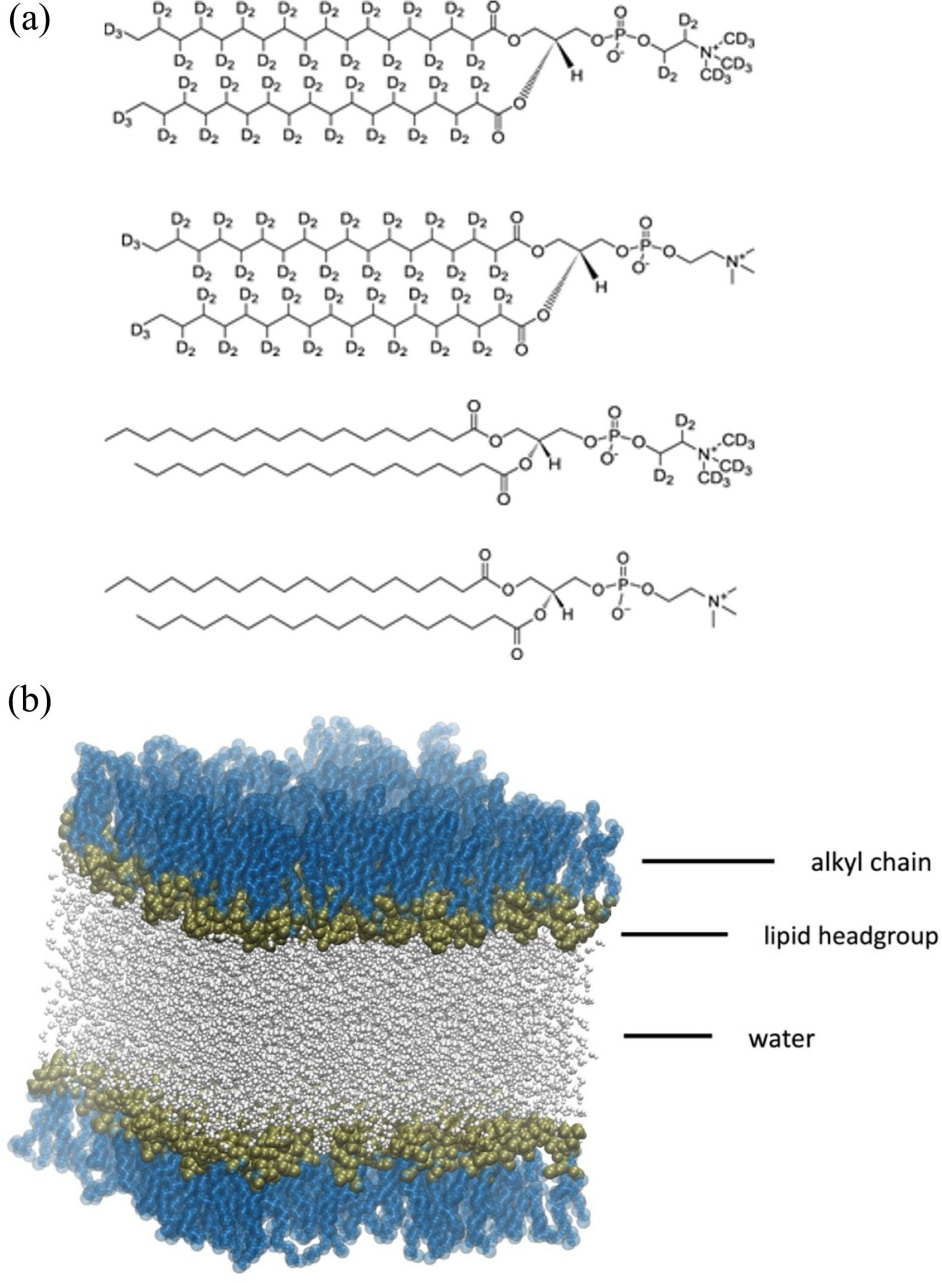
(*a*) Atomic structure of L-α-distearoylphosphatidylcholine (DSPC) shown with the four deuteration schemes. From top to bottom, these are D-83-DSPC (fully deuterated), D70-DSPC (tail deuterated), D13-DSPC (head deuterated) and H-DSPC (hydrogenated). The first three were measured on subphases of D_2_O or air-contrast matched water (ACMW—8.1% D_2_O/91.9% H_2_O), while H-DSPC was measured on D_2_O only, leading to seven contrast measurements in total. (*b*) Snapshot of the simulation cell, containing two DSPC monolayers arranged normal to the Z axis which sandwich a water layer between the respective headgroups. The hydrophobic tails are separated, via the periodic boundary conditions of the simulation cell, by a vacuum layer. Each monolayer is composed of 100 DSPC molecules.

The data used in this study were first described in [38], and a more detailed description of their collection can be found there. But briefly, L-α-DSPC was dissolved in chloroform to a concentration of 2 mg ml^−1^. Monolayers were then spread at the air–water interface on a Langmuir trough, mounted on the CRISP reflectometer at the ISIS pulsed neutron source. The trough was either filled with D_2_O or air-contrast matched water (ACMW, 8.1% D_2_O/91.9% H_2_O), and one of four choices of lipid deuteration measured (figure 1a), namely, fully deuterated (D83-DSPC), head deuterated (D13-DSPC), tail-only deuterated (D70-DSPC) or hydrogenated (H-DSPC), leading to seven deuteration combinations in total (see top of electronic supplementary material, table S1). Each monolayer was measured at four pressures; 20, 30, 40 and 50 mN m^−1^, over a momentum transfer range of 0.01–0.6 Å^−2^.

### 2.3. Simulations

We simulated a double monolayer system in which a water layer is sandwiched between the headgroups of two monolayers arranged normal to the Z axis (see figure 1b). Each monolayer is composed of 100 DSPC molecules. The system was built with CHARMM-GUI [39] and parametrized using the CHARMM36 force field for DSPC [40] and TIP3P for water [41]. Note that we did not use the recent LJ-PME correction for CHARMM36 phospholipids [42]; see §4. In accordance with the experiment, no ions were added to the water layer. All simulations were performed using NAMD [43] and a timestep of 2 fs. Temperature was kept constant with a Langevin thermostat, and for cases in which the isotropic or anisotropic pressure was fixed, a Nose–Hoover barostat was used. The long-range interactions were treated with a particle-mesh Ewald (PME) method [44] with a grid width of 1.0 nm, and a smooth cut-off distance of 10 Å was used to calculate the Lennard–Jones interaction and the real space part of the Ewald sum. To model the full isotherm, we need to both compress and expand the system in a stepwise fashion to give lower and higher APL values, and we investigated two schemes to do this.

In the first scheme, we ran an initial unbiased equilibration at a temperature T = 298 K and isotropic pressure *p* = 1 atm, resulting in monolayers with APL 49.3 Å^2^ and a linear dimension of approximately 70 Å along the X and Y axes. We then expanded or compressed gradually by applying an outward or inward pressure in the X–Y plane, while keeping the pressure along Z constant. The lateral pressure was adjusted in steps of 10 dyn cm^−1^ and the simulation runs for 10 ns each time. The pressure was increased until the desired APL, differing by about 1 Å^2^ compared with the previous point, was reached. We then fixed the APL and simulated in an NPAT ensemble for 50 ns, in which X and Y can vary as long as the area remains the same and a constant pressure of 1 atm is applied in the Z direction. Each simulation was then extended by 1 ns to record the surface tension, using an NVT ensemble to avoid length changes along the Z axis. The pressure components for each 1 Å slice of the system along Z were extracted and used to calculate the surface tension as


(2.1) $$\gamma =\frac{L_{z}}{2}*\left[P_{zz}-\frac{1}{2}\left(P_{xx}+P_{yy}\right)\right],$$

where *L_z_* is the length of the simulation box along the Z axis perpendicular to the membrane, while *P_xx_*, *P_yy_* and *P_zz_* are the diagonal components of the pressure tensor, outputted by the ‘PressureProfile’ option in NAMD. The surface pressure is then given by the standard relation Π= γ(aw) − γ(m), where γ(aw) is the surface tension of the water–vacuum interface, and γ(m) is the calculated surface tension of the monolayer.

In the second scheme, we follow the semi-permeable box method of de Souza *et al.* [45]. Starting from the system previously built in CHARMM-GUI, we extend the water layer up to 23 Å each side of the X axis, and virtual walls are introduced normal to the axis, through which water but no lipids can pass (electronic supplementary material, figure S1a). These virtual walls are realized by an external potential that acts on lipid molecules only, creating an inward force on the monolayer. The surface tension can then be estimated as γ = <*Fwall>/4L_Y_*, where <*Fwall>* is the mean force exerted on the molecules by the virtual walls during the simulations, and *L_Y_* is the length of the simulation box along the Y axis which is fixed at the original length from the system build of 91 Å. To equilibrate the system, the inner walls were placed initially at x = ±60 Å for 50 ns and then moved stepwise every 10 ns to x = ±45, ±40, ±35 and ±30 Å, corresponding to APL of 81.9, 72.8, 63.7 and 54.6 Å^2^, respectively. Then, to assess an APL range comparable with the first method, we continued shifting the walls stepwise every 10 ns, moving them closer to each other by 1 Å each side of the X axis, up to x = ±23 Å. The simulations are performed in the NVT ensemble (20 ns for each APL) to preserve the excess of water along X necessary to the surface tension calculation. A variation of this scheme was also applied, based on a semi-empirical correction [46]. In particular, the surface tension values calculated from the simulation are multiplied by a coefficient based on the thickness of the region, for each simulation, where the density of water along the direction normal to the interface drops from 90 to 10% of the maximum density.

For both schemes, the MD trajectories were used to calculate average number density (ND) profiles along the Z axis by averaging across successive slices in the X–Y plane with a thickness of 0.5 Å. This was done with custom scripts built upon the Made2reflect library [47]. Since the simulation cell contains two copies of the monolayer, placed on either side of Z = 0, we averaged their ND values and reported a combined profile.

### 2.4. Calculation of neutron reflectivity from simulations

A simulated neutron (or X-ray) reflectivity curve is calculated from the variation in SLD across an interface [48]. The SLD at a given point is obtained from the number of atoms of each type per unit volume, multiplied by the bound coherent scattering cross-section of each particular element


(2.2) $$\rho \left(z\right)=\;\sum_{i}\frac{n_{i}b_{i}}{V},$$

where *b_i_* is the scattering cross-section of element *i*, *n_i_* is the number of atoms and *V* is the volume. To calculate the SLD from a simulation, the key output to be obtained is the ND ($n_{i}/V)$ of each constituent element of the system. Reflectivity averages over the X–Y plane, and so the distributions required are the average atomic compositions in the Z direction, normal to the interface. To obtain these distributions, the simulation box is first split into a number of slices along the Z axis, and the number of each element type is counted and divided by the volume of the slice [19,20], as described in the previous section.

As is common with NR, we perform a simultaneous analysis of a number of deuterations (conventionally known as contrasts), and so some additional care is required in the treatment of hydrogen isotopes. To model the contrasts given in figure 1a, we consider the hydrogen number densities of the tails and the heads separately and then multiply them by the cross-section of either hydrogen or deuterium as appropriate for each contrast. We convert the water distributions directly into volume fractions, since it is clear that the ND of water should vary from unity in the bulk phase to 0 on the vacuum side of the monolayer, and it is thus trivial to normalize these distributions. These fractions can then be directly multiplied by the bulk SLD value of the solvent, which we include as fitting parameters.

Some additional work is required in order to complete the model, since all Langmuir monolayers are subject to capillary waves of the water surface [49], the effect of which is to smear the SLD profile when averaged over the experimental neutron beam. We do this by convoluting the whole model with a Gaussian, as we have shown previously in dealing with fluctuating membranes [18]. The width of the Gaussian is a fitting parameter of the model, termed the substrate roughness. Additionally, we include a scale factor as usual when analysing neutron reflection from monolayers, accounting for the attenuation of the neutron beam caused by the smaller slits necessary when measuring the direct reference beam as compared with the reflected measurement in these types of measurement. Finally, we fit a constant background to each measurement to account for incoherently scattered neutrons. The backgrounds in monolayer experiments arise from bulk scattering and are largely dependent on hydrogen loading of the bulk, so we use a separate parameter for those contrasts measured on D_2_O as opposed to those measured on ACMW.

So, given ND profiles from a simulation, a total of six additional fitting parameters need to be determined: substrate roughness, scale factor, background D_2_O, background ACMW and the SLDs of the two bulk phases. The seven contrasts measured at each pressure were fit simultaneously (i.e. with the overall goodness of fit $\chi^{2}$ taken as the sum of the individual $\chi^{2}$ from each contrast) using the Matlab API for the RAT toolbox [50]. For each contrast, the reduced chi-squared is defined as


(2.3) $$\chi_{contrast}^{2}=\;\frac{1}{\left(N-P\right)}\sum_{i}\frac{{\left[R_{exp}\left(q_{i}\right)-\;R_{sim}\left(q_{i}\right)\right]}^{2}}{{\left[\delta R_{exp}\left(q_{i}\right)\right]}^{2}},$$

where *N* is the number of data points, *P* is the number of fitting parameters, $R_{exp}\left(q_{i}\right)$ is the experimental intensity, $R_{sim}(q_{i})$ is the modelled reflectivity intensity derived from the simulations and a given set of experimental parameters, and $\delta R_{exp}(q_{i})$ is the experimental Poisson counting statistical error at each point. Each fit is over the sum of the chi-squared values for each individual contrast,


(2.4) $$\chi_{Total}^{2}=\sum \chi_{contrast}^{2}.$$

For each simulation, the disposable experimental parameters (backgrounds, scale factor, interfacial capillary roughness and bulk SLDs) were optimized to a best fit via a Bayesian analysis using the Paramonte sampler [51] using 50 000 Markov chain Monte Carlo (MCMC) steps. From the resulting Markov chain, marginalized parameter posteriors were calculated from the last 10 000 steps of the chain [52], with the maxima of the posterior distributions taken as the best-fit parameters, and their widths used to determine their confidence intervals.

## 3. Results

### 3.1. Simulations

**Figure 2.**
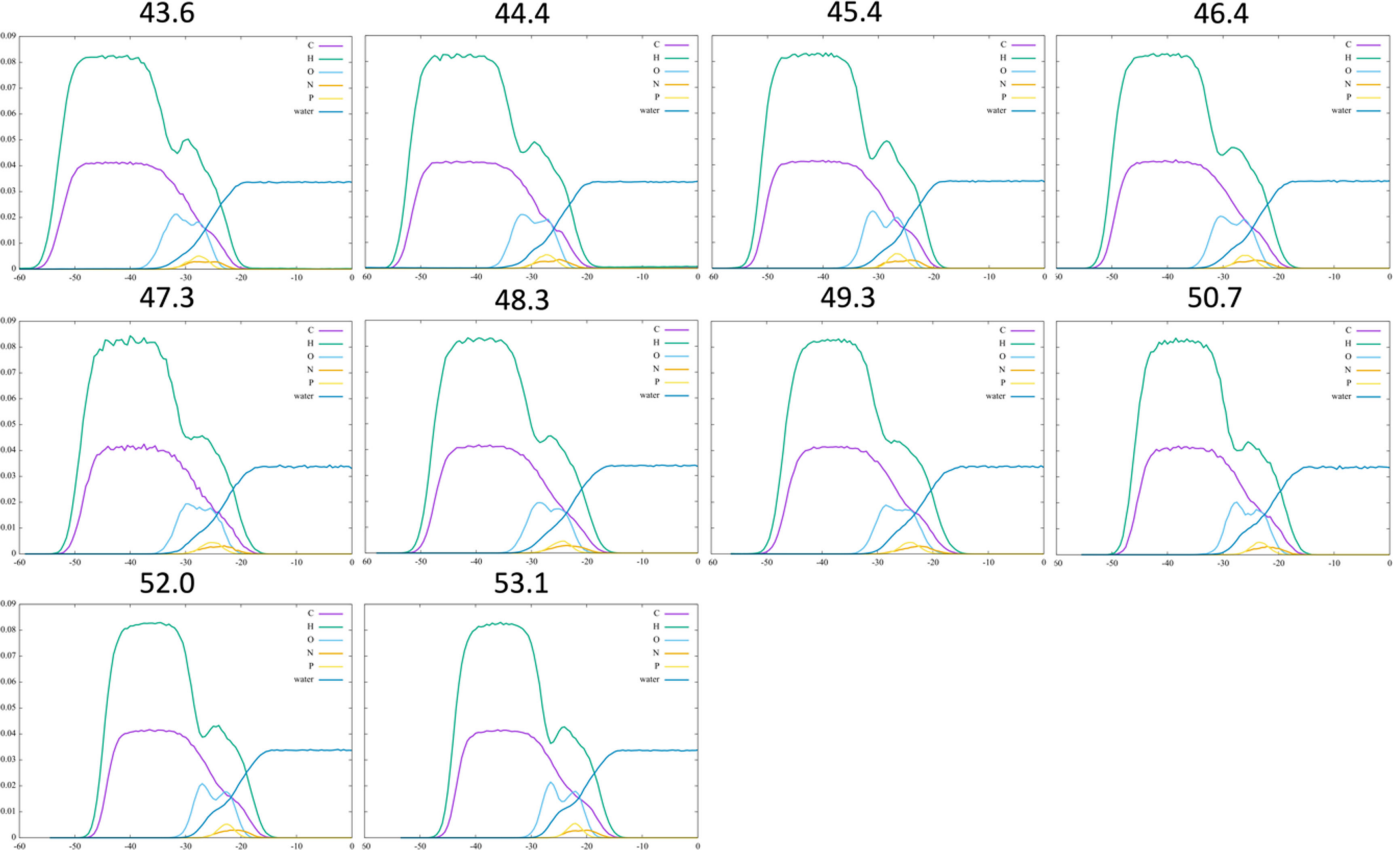
Per-element number density profiles of DSPC monolayer, as calculated from MD simulations using compression and expansion from an initial equilibrated structure with area per lipid (APL) of 49.3 Å^2^. The APL for each simulation is indicated above the respective panel (in Å^2^).

In the first compression scheme, we simulated the DSPC monolayer system for 50 ns in the NPAT ensemble at the equilibrated APL of 49.3 Å^2^, and at a series of nine additional APL values (43.6, 44.4, 45.4, 46.4, 47.3, 48.3, 50.7, 52.0 and 53.1 Å^2^) obtained by compressing and expanding the monolayer (see §2). The corresponding ND profiles per element along the Z axis are reported in figure 2. The profiles remain qualitatively similar, due to the lack of any phase transition, but there is a gradual broadening as the APL is reduced. At each APL, we estimated the surface tension of the monolayer (electronic supplementary material, figure S2a). By using γ(aw) = 55 mN m^−1^, as done in [53] and which is typical of theoretical water models [52], we obtain the orange isotherm in electronic supplementary material, figure S2b, while the blue isotherm is obtained with the typical experimental value γ(aw) = 72 mN m^−1^ [54].

In the second scheme, starting from the equilibrated system at APL = 63.7 Å^2^ (walls placed at x = ±35 Å), we then moved stepwise to x = ±30, ±29, ±28, ±27, ±26, ±25, ±24 and ±23 Å. In this case, the APL was calculated from the distance between the inner walls along X multiplied by the cell dimension along Y, then divided by the number of lipids (100 per leaflet). The resulting systems have APL = 54.6, 52.8, 50.96, 49.1, 47.3, 45.5, 43.7 and 41.9 Å^2^, respectively. A simulation time of 20 ns per APL turned out sufficient to converge the surface tension calculations (electronic supplementary material, figure S1b).

On the other hand, while this calculation is much faster compared with the first scheme, it is based on a stepwise placement of the virtual walls rather than a gradual compression of the simulation box. We observe that this does not allow for an accurate reproduction of the number densities of the phospholipid chain, when compared with standard compression (electronic supplementary material, figure S3). In particular, the headgroup phosphorus and oxygen profiles significantly fluctuate, consistent with the monolayer curvature observed by Prabhu *et al*. [46]. For this reason, we do not use these number densities for fitting the reflectivity data.

For every simulation with walls placed from ±30 to ±23 Å, we also calculated the density profile of water. Following the procedure described in [46], we used these profiles to define the width of the region Lz where the water density drops from 90 to 10% of the maximum density. All these values, as well as the one extracted from the first wall placement (±45 Å) as a reference for low surfactant surface coverage, were plotted and fitted with an exponential function (electronic supplementary material, figure S4a). The corrected surface pressure is then obtained by multiplying by a coefficient that is the ratio between the reference value of Lz and each fitted value. The result of this procedure is reported in electronic supplementary material, figure S4b.

These simulations yield isotherms for the DSPC monolayer, with APL fixed by the simulation cell, and the surface pressure estimated from the first scheme or the second scheme (with or without correction). It can be immediately seen that there is a large (approx. 10-fold) discrepancy between the different theoretical methods (electronic supplementary material, figures S2 and S4). We discuss this further below, and in comparison with the experimental and fitted isotherms.

### 3.2. Experiments

**Figure 3.**
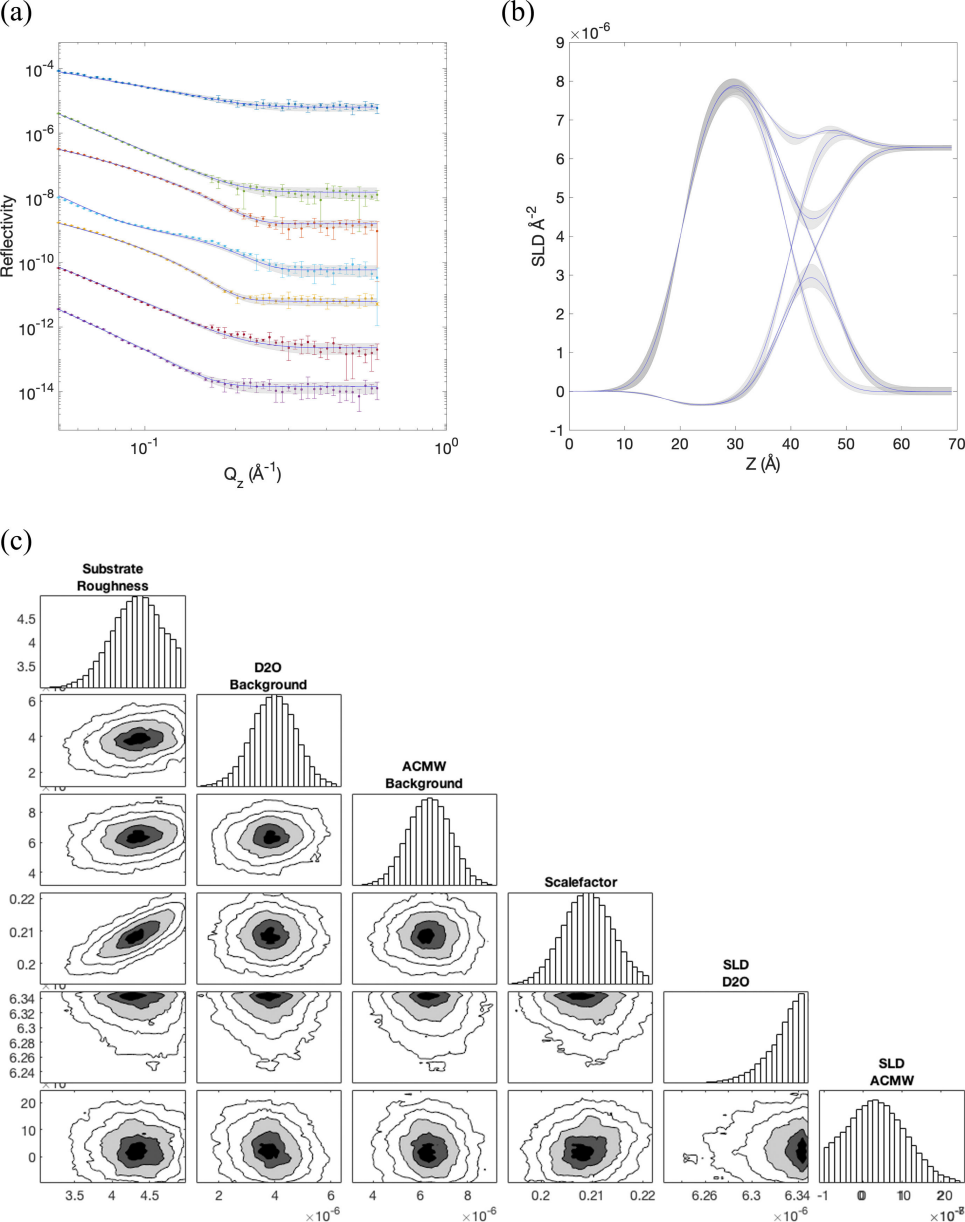
Fit of the atomistic monolayer model simulated with an APL of 50.7 Å^2^ to the NR data collected at a surface pressure of 20 mN m^−1^. (*a*) The best-fit curves for the seven contrasts after refinement of the additional fitting parameters, along with the 95% CIs. (*b*) The corresponding scattering length density (SLD) profiles. (*c*) Posterior distributions of the six fitting parameters to the fit in (*a*), along with the corner plot of parameter correlations. The six parameters are substrate roughness, a constant background for measurements in D_2_O solvent or air-contrast matched water (ACMW), a scale factor accounting for attenuation of the neutron beam, and the bulk SLDs of D_2_O solvent and ACMW.

Reflectivity datasets were collected at four surface pressures (20, 30, 40 and 50 mN m^−1^), for each of seven deuteration combinations (see §2). Each simulation carried out at a fixed APL can be used to fit the set of contrasts at a given experimental pressure, optimizing the six additional fitting parameters in each case. As a representative example, figure 3 shows the fit to the 20 mN m^−1^ dataset using the simulation carried out at 50.7 Å^2^. Figure 3a shows the best fit curves for the seven contrasts, along with the 95% CIs, which correspond to the SLD profiles shown in figure 3b. In the SLD profiles, the air bulk phase is at the extreme left of the plot, whilst the aqueous bulk phase is on the right. Between approximately 20 and 40 Å, the lipid tails have an SLD of either *ca* 8.2 × 10^–6^ Å^−2^ for deuterated tails, or *ca* −0.4 × 10^–6^ Å^−2^ for the hydrogenated case. Similarly, in the head region between approximately 40 and 55 Å, the head SLDs take higher values for deuterated heads rather than the hydrogenated equivalent. Note that the SLD profiles appear broader than the ND profiles shown in figure 2, due to the convolution with the modelled substrate roughness. Finally, at the extreme right of the plot, the bulk phases take on either the SLD of D_2_O (approx. 6.35 × 10^−6^ Å^−2^) or that of ACMW (close to 0).

The SLDs of the bulk phases, along with the scale factor, substrate roughness and the backgrounds are fitting parameters, as described in §2, and electronic supplementary material, table S1 gives the values of all six fitting parameters for each combination of experimental data and simulation. Figure 3c shows typical posterior distributions of the six fitting parameters for the case shown in figure 3a, along with the corner plot of parameter correlations, showing that the parameter posteriors are all more or less Gaussian and uncorrelated. The exception, however, is the significant positive correlation between the scale factor and the substrate roughness, which is typically seen for reflectivity monolayers at the air–water interface [52], and that the same correlation is seen in these results gives confidence in the fitting procedure. The correspondence between simulation and experiment is excellent in this case, with an overall $\chi^{2}$ of 30.3 (note that this value is summed over the seven measured contrasts, and the individual $\chi^{2}$ values for each contrast at each pressure are shown in electronic supplementary material, table S2).

**Figure 4.**
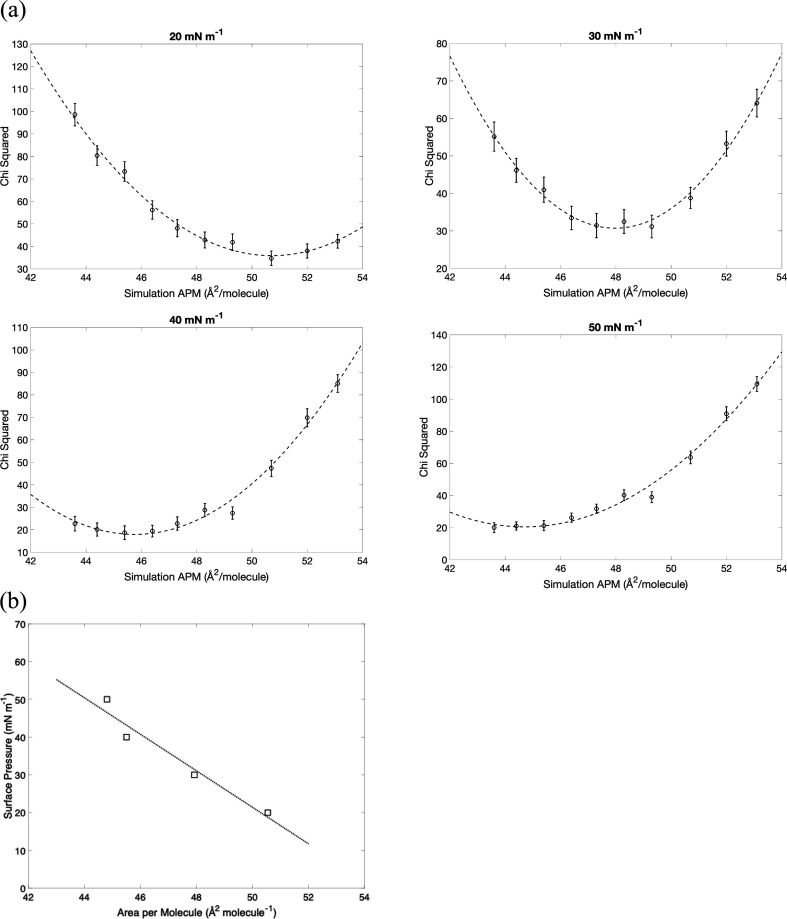
(*a*) For each experimental surface pressure measured, a plot of the *χ*^2^ goodness of fit (equation (2.4)) using the simulation output against the fixed APL used in the simulation. The dashed lines are quadratic fits. (*b*) A plot of the surface pressure against APL, using values for the latter taken from the minima of the curves in (*a*).

At each of the four surface pressures, the experimental data were compared against the whole compression series of simulations (using the first scheme), and the best fit of $\sum \chi^{2}$ for each simulation area is listed in electronic supplementary material, tables S1 and S2, and plotted in figure 4a. At each pressure, the quality of the fit varies considerably with assumed APL, reaching a minimum where the area of the simulation gives a structure which best matches the structure of the monolayer measured in the neutron experiment. The area chosen for the simulation is critical to the quality of the fit, with the worst fit having twice the $\sum \chi^{2}$ of the best in each case. The lines in the plots are third-order polynomial fits, and it will be noted that the minimum point of the fitted curve shifts to lower areas as the pressure is increased.

### 3.3. Isotherms and structural analysis

**Figure 5.**
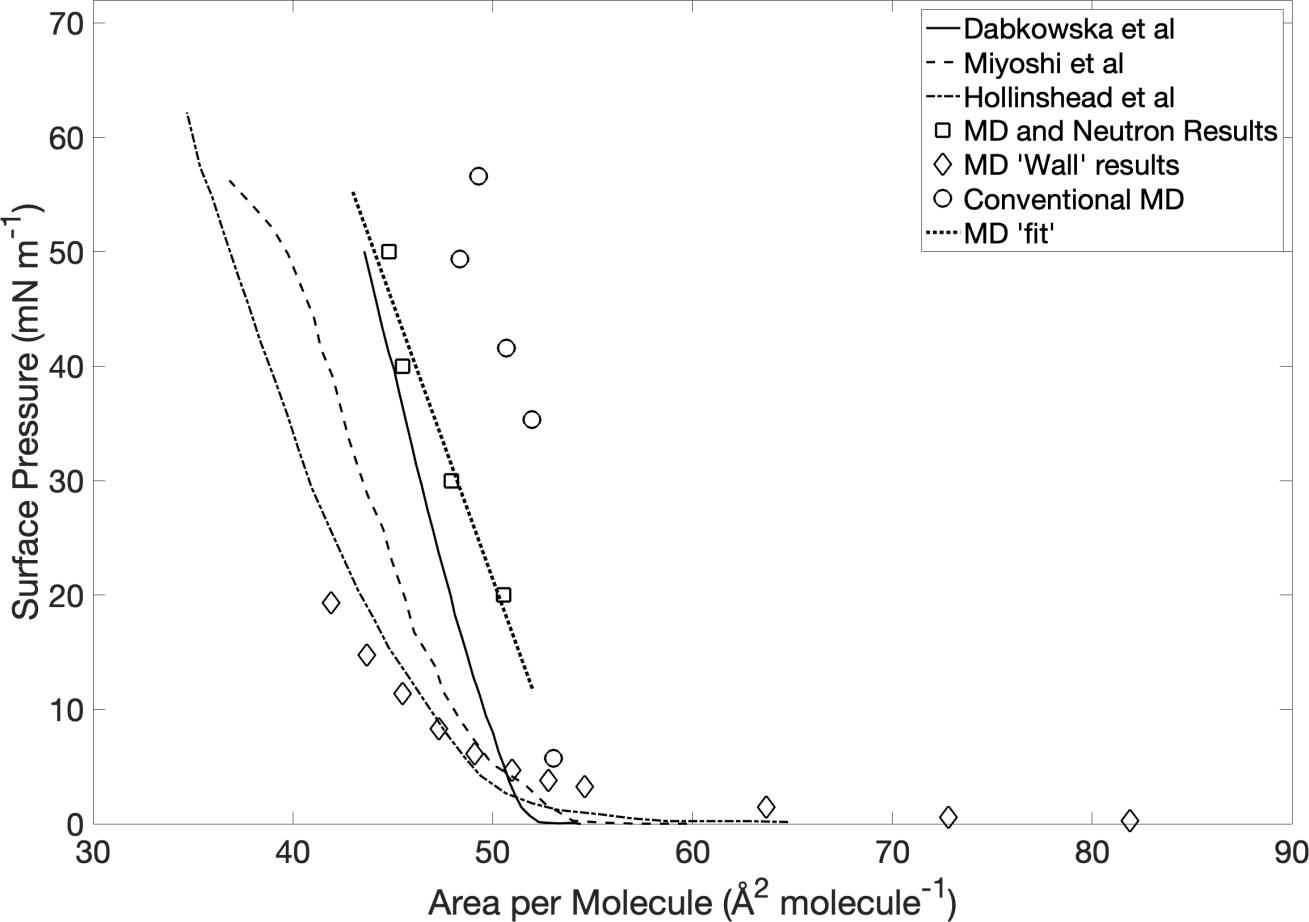
Isotherms of surface pressure against APL for a range of experimental and theoretical approaches. The curves show three experimental isotherms taken from the literature. Conventional MD refers to the surface pressure calculated using the first scheme, assuming an air/water surface tension *γ*(aw) = 55 dyn cm^−1^. MD Wall refers to the uncorrected surface pressure from the second scheme. MD and Neutron refer to the results of the fitting procedure, also shown in figure 4b.

In a monolayer experiment, the isotherm is always collected, and in principle, the APL associated with each measurement should be experimentally available from the known amount of sample spread and the area of the trough. In practice, however, there are considerable uncertainties in this procedure. Figure 5 shows three representative published isotherms for pure DSPC [38,55,56], and there is significant variation between them. The lift-off areas of the isotherms are very similar in each case, but the gradients vary considerably, leading to a difference of nearly 10 Å^2^ per molecule at high pressures (40–50 mN m^−1^) between the most dissimilar cases. If this is representative of the variability inherent in the measurement of monolayers, then simply relying on the experimental isotherm to provide an estimate of the APL for the simulation would not be reliable.

The minima from the fitted polynomials in figure 4a show that the APL at the minimum decreases with increasing experimental pressure, allowing us to recover an isotherm from the fit to the neutron data. These optimal values for the area are plotted in figure 4b and superimposed on the isotherms in figure 5. The isotherm recovered from the neutron fits gives higher APLs for a given surface pressure than the experimental isotherms but is relatively close to that of Dabkowska *et al*. [55].

For a given APL, the surface pressure can also be estimated from the simulation, following the two schemes described in §2. In comparison with the experimental and fitted isotherms (figure 5), the conventional compression method (first scheme) overestimates the surface pressure while the walls method (second scheme) underestimates it. As mentioned above, there is a large uncertainty in the estimate from simulation, depending on the method used.

**Figure 6.**
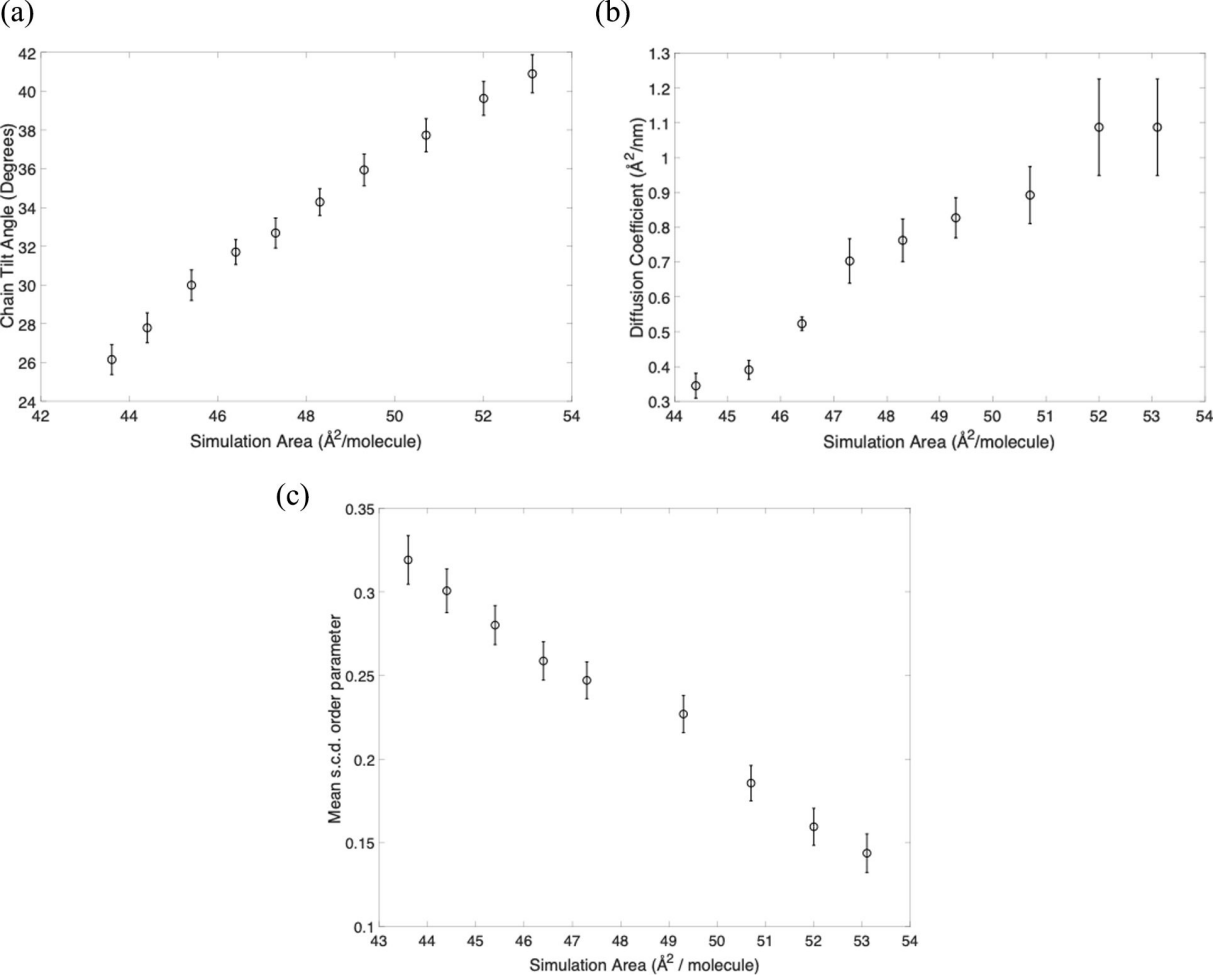
Structural and dynamic parameters derived from simulation as a function of the fixed APL. The lipid tail tilt angle (a) and the self-diffusion coefficient (b) are averages over all lipids in the monolayer and over the last 10 ns of the trajectory. The S_CD_ deuterium order parameter (c) is averaged over carbons C4–C16 of the acyl tails. The full structural data are plotted in electronic supplementary material, figure S5.

Finally, we consider the consequences of the APL estimation for a structural interpretation of the sample. In electronic supplementary material, figure S5, we plot the deuterium order parameter S_CD_, the average tilt angle of the lipid tail, and the lipid self-diffusion coefficient for the series of simulations at different fixed APLs. The trends are shown in figure 6, showing that for higher APL, as expected, the tail becomes less ordered and more tilted. In addition, the mobility of individual lipids, as quantified by the self-diffusion coefficient, increases as the area available rises. Thus, determining the optimal APL not only improves the fit to experimental data but also changes the structural and dynamic properties of the derived model.

## 4. Discussion

Traditionally, the isotherm from a film balance is considered a unique measurement of the surface thermodynamics of a particular system, but it is not always appreciated that the precise details of the measurement can strongly influence the isotherm itself. Even though diligent users will often screen their own isotherms for reproducibility, comparisons with the isotherms of others are rare. One such comparison has been reported for DPPC (dipalmitoylphosphatidylcholine) monolayers for a large set of published isotherms [57], which showed large variabilities in area comparable to (or greater than) our own comparison for DSPC. Isotherms can change as a function of temperature, compression rate, ionic strength of the subphase, contaminants, the concentration and solvent used for the spreading solution, or even the geometry of the trough, and the authors suggested combinations of these to account for the discrepancies found [57].

The generalized phase diagram for isotherms of saturated lipid monolayers can reveal distinct phases when taken over an appropriate range of pressure and area [57,58]. At large areas and low pressures, a diffuse and less condensed ‘liquid expanded’ (LE) phase may be seen. In the LE phase, the lipid tails are loosely packed and have significant configurational freedom, and the LE phase is often considered analogous to the fluid phase of lipid bilayers. At lower areas, a tightly packed and more ordered liquid condensed (LC) phase may be found. Not all lipids, however, show the full range of phases, depending generally on chain length and temperature, and lipids that would be expected to be in the fluid phase at room temperature stay in the LE phase of the isotherm at all pressures (e.g. the short chain (14 : 0) lipid DMPC - Dimyristoylphosphatidylcholine). Conversely, for room temperature gel phase lipids such as DSPC the opposite is true, and the isotherm is generally assumed to remain in the tightly packed LC phase (as is clear from an example simulation snapshot of DSPC simulations shown in electronic supplementary material, figure S6). The intermediate DPPC transitions between phases on compression showing a clear phase boundary and the fact that DSPC remains in a single phase along the isotherm simplify the analysis for the purposes of this article as no account of the phase boundary has to be made in the analysis.

However, for LC phase monolayers, the idealized continuous and homogeneous monolayer spread across the entire experimental trough is unlikely, and instead a mosaic of LC domains is expected, the distribution and organization of which would vary with the details of each monolayer preparation. Using Brewster angle microscopy, Jyoti *et al.* [59] saw that domain structure in DPPC monolayers is highly dependent on compression rate, and using fluorescence microscopy Klopfer and Vanderlick [60] came to similar conclusions. For DSPC, Dabkowska *et al.* observed that at very low pressures (i.e. at the lift-off area of the isotherm) individual domains of condensed phase material spontaneously formed, which retained their distinct identity during compression, never merging into one continuous phase [55]. Such a heterogeneous layer could be expected to differ between preparations in terms of domain size and organization, and this may go some way to explaining the experimental differences in isotherms seen by different workers. In a neutron reflection experiment, however, the beam illuminates a large area of the interface and is collected over the timescale of many minutes (sometimes hours), making the data both a temporal and spatial average of the surface. So in contrast to the isotherm, the precise details of the domain structure should not be expected to affect the overall neutron signal.

The fits of the neutron data to simulations shown in figure 4a can be used to construct an independent isotherm when fit by simulations, by plotting the minima of the quadratic fits in figure 4a against experimental surface pressure, as shown in figure 4b. This fitted ‘MD isotherm’ implies higher APL at a given surface pressure than any of the experimental isotherms (figure 5), and indeed it could be argued that any experimental isotherm is likely to underestimate the APL, since all lipids are at least partially soluble in water, and there is also often material loss at the interface between the barriers and monolayers in a Langmuir trough [61]. If the actual quantity of lipid in the monolayer is lower than expected, then experimental APL, which is calculated from the spread amount divided by the trough area will be an underestimate.

In terms of simulated isotherms, the isotherms we have derived purely from simulation—using either of two methods of calculating the surface tension—do not agree with each other or with any of the experimental isotherms. This is consistent with a similar picture obtained for DPPC [53], where it was observed not only that reproduction of experimental curves can be problematic in most force fields, but also that most of the all-atom models produce large negative values of the surface tension at low APL (which is also the case for this work, as shown in electronic supplementary material, figure S2).

There have been attempts to improve the areas obtained from simulations. For example, an LJ-PME correction for CHARMM36 phospholipids [42] has been recently proposed, which shows a claimed improvement in the predicted APL for monolayers. Our approach is different in philosophy to this, in that we constrain the APL of the simulations via agreement with experimental data, rather than by aiming to match it solely from the force field. This compensates to some extent for any deficiencies which may exist in the force field but also any other inaccuracies in the modelling. After all, the best information available about the real system is the direct (although coarse) reflectivity measurement of the atoms per unit volume of the sample itself, and adjusting the simulation to match this data gives the best chance of obtaining a simulation that best corresponds to the actual monolayer.

In terms of modelling the reflectivity data, using an MD simulation to model the reflectivity from a monolayer is a very expensive way of going about it, and there are other far quicker methods of data analysis available. There are models available of increasing sophistication, which roughly in sequence go from crude traditional ‘layer models’ parametrized solely in terms of thickness, SLD and roughness; to layer models which use known component volumes to parametrize layers in terms of more realistic parameters such as molecular areas; and finally, methods which mimic the distributions groups of atoms directly using Gaussians or other functions. Each of these is a far quicker way of ‘fitting’ reflectivity data than performing an atomistic simulation, and—particularly in terms of Gaussian pseudomolecular groups—could be expected to match the data as least as well as simulations and give equivalent values of chi-squared. The point of using MD to analyse reflectivity data then is not to try to get a better fit (although it will improve over layers however they are parametrized), but to try to gain a deeper insight into the physical properties of the system being investigated, by using the atomistic simulations to extract physical insights which are not available from the reflectivity data directly.

However, any insight gained from this atomic detail can be wildly misleading if there is an incorrect assignment of key experimental variables (in this case the APL). To illustrate this, in figure 6, we show some quantities derived from the MD simulations which might be useful in some following biophysical analysis, namely, the chain tilt angle, the diffusion coefficient, and the meanS_CD_ order parameter (which is a measure of the degree of motional disorder of the chains) over the entire range in area of our simulations. From the lowest area to the highest, the chain tilt varies by approximately 15 degrees, the S_CD_ order parameter approximately halves, and the diffusion coefficient changes by a factor of four.

This means that using an incorrect APL could lead to in incorrect further analysis, indicating the problem with taking the experimental isotherm for the sample area. Taking the experimental surface pressure of 20 mN m^−1^ as an example, we can compare the inferred structural and dynamic properties of the monolayer for an APL of 45.4 Å^2^ (from the middle of the three experimental isotherms) or an APL of 50.5 Å^2^ (from the results of the MD fit). At these surface pressures, the average tilt angle would be interpreted as being approximately 28^o^ (isotherm) versus approximately 30^o^ (MD), the S_CD_ order parameter would be 0.3 (isotherm) versus 0.175 (MD), and the diffusion coefficient would be approximately 0.35 (isotherm) versus approximately 0.8 (MD). Although we *can* fit a system with APL of 45.4 Å^2^ to the data, the fit is worse (as judged by the $\chi^{2}$ in figure 4 and electronic supplementary material, table S2), and the inferred structural and dynamic properties are significantly different. These discrepancies will take on more significance as one moves to functional systems such as lipid–peptide mixtures, or proteins interacting with pre-spread lipid films from solution for example.

In a single-component system, such as the pure monolayer considered here, the only restraint on the simulation that we consider is the APL. This leads to the one-dimensional plots of $\chi^{2}$ versus restraint shown in figure 4, from which the optimal value of the restraint is easy to determine by a simple quadratic fit. For more complex investigations, multiple restraints may be appropriate, e.g. for self-assembled monolayers (SAMs) it may be appropriate to restrain and optimize the chain tilt or the packing density with respect to the substrate surface in addition to the APL. It may also be useful in some circumstances to optimize variables such as temperature and pH (or degree of protonation) to achieve the best agreement with the experimental system. In such cases, one needs a multi-variate fit that is easily tractable with modern optimization methods. Nevertheless, the principle remains the same, in that the values of restraints applied in MD should be chosen based on the correspondence between simulations and experimental data, otherwise incorrect conclusions regarding the behaviour of the systems could be drawn.

## 5. Conclusions

We have investigated the analysis of reflectivity data using atomistic MD simulations. We find that the correct choice of APL used in the simulations is crucial to the analysis. Changes in the lateral APL imply changes to the ND profiles in the perpendicular direction, and the fit to reflectivity data is very sensitive to the latter. We propose a new approach in which the optimal APL is inferred from the fit-to-reflectivity data, rather than being taken from experimental isotherms which are known to be variable. Optimizing the structural model not only improves the fit to the measured data, but also affects the values of derived structural and dynamical quantities, and so may have implications for interpretations drawn.

## Data Availability

Bespoke scripts for post-processing MD trajectories and generating number density profiles are available in Github https://github.com/valerialosasso/scripts_MD_NR and archived within the Zenodo repository at [62]. This repository also contains all the relevant neutron reflectivity data presented in the paper. Molecular dynamics data have been deposited in Zenodo [63]. For the first compression scheme, individual zip files are named after the fixed APL and contain the trajectory and the topology file. For the second compression scheme, a single zip file contains the full set of trajectories and the topology file.

## References

[1] Fragneto G, Charitat T, Daillant J. 2012 Floating lipid bilayers: models for physics and biology. Eur. Biophys. J. **41**, 863–874. (10.1007/s00249-012-0834-4) 22825799

[2] Lakey JH. 2019 Recent advances in neutron reflectivity studies of biological membranes. Curr. Opin. Colloid Interface Sci. **42**, 33–40. (10.1016/j.cocis.2019.02.012)

[3] Hughes AV, Patel DS, Widmalm G, Klauda JB, Clifton LA, Im W. 2019 Physical properties of bacterial outer membrane models: neutron reflectometry & molecular simulation. Biophys. J. **116**, 1095–1104. (10.1016/j.bpj.2019.02.001) 30850116 PMC6428969

[4] Clifton LA *et al*. 2015 An accurate in vitro model of the E. coli envelope. Angew. Chem. **54**, 11952–11955. (10.1002/anie.201504287) 26331292 PMC4600229

[5] Giner-Casares JJ, Brezesinski G, Möhwald H. 2014 Langmuir monolayers as unique physical models. Curr. Opin. Colloid Interface Sci. **19**, 176–182. (10.1016/j.cocis.2013.07.006)

[6] Verreault D, Hua W, Allen HC. 2012 From conventional to phase-sensitive vibrational sum frequency generation spectroscopy: probing water organization at aqueous interfaces. J. Phys. Chem. Lett. **3**, 3012–3028. (10.1021/jz301179g) 26292243

[7] Hoernke M, Schwieger C, Kerth A, Blume A. 2012 Binding of cationic pentapeptides with modified side chain lengths to negatively charged lipid membranes: complex interplay of electrostatic and hydrophobic interactions. Biochim. Et Biophys. Acta **1818**, 1663–1672. (10.1016/j.bbamem.2012.03.001) 22433675

[8] Rojewska M, Smułek W, Kaczorek E, Prochaska K. 2021 Langmuir monolayer techniques for the investigation of model bacterial membranes and antibiotic biodegradation mechanisms. Membranes **11**, 707. (10.3390/membranes11090707) 34564524 PMC8471293

[9] Clemente-León M, Coronado E, Soriano-Portillo A, Martín-Romero MT, Pérez-Morales M, Domínguez-Vera JM, Gálvez N. 2007 Langmuir monolayers and Langmuir–Blodgett films of ferritin prepared by using a surfactant mixture of eicosylamine (EA) and methyl stearate (SME). Polyhedron **26**, 1871–1875. (10.1016/j.poly.2006.09.022)

[10] Mogilevsky A, Jelinek R. 2011 Gold nanoparticle self-assembly in two-component lipid Langmuir monolayers. Langmuir **27**, 1260–1268. (10.1021/la103718v) 21050012

[11] Xue Z, Xue N. 2019 The cooperative effect of BSA Langmuir monolayers and magnesium ions on calcium carbonate crystallization. OALib **06**, 1–8. (10.4236/oalib.1105551)

[12] Qian S, Sharma VK, Clifton LA. 2020 Understanding the structure and dynamics of complex biomembrane interactions by neutron scattering techniques. Langmuir **36**, 15189–15211. (10.1021/acs.langmuir.0c02516) 33300335

[13] Kinnun JJ, Scott HL, Ashkar R, Katsaras J. 2021 Biomembrane structure and material properties studied with neutron scattering. Front. Chem. **9**, 642851. (10.3389/fchem.2021.642851) 33987167 PMC8110834

[14] Fragneto G. 2012 Neutrons and model membranes. Eur. Phys. J. Spec. Top. **213**, 327–342. (10.1140/epjst/e2012-01680-5)

[15] Lu JR, Lee EM, Thomas RK. 1996 The analysis and interpretation of neutron and X-ray specular reflection. Acta Crystallogr. Sect. Found. Crystallogr. **52**, 11–41. (10.1107/s0108767395011202)

[16] Schalke M, Lösche M. 2000 Structural models of lipid surface monolayers from X-ray and neutron reflectivity measurements. Adv. Colloid Interface Sci. **88**, 243–274. (10.1016/s0001-8686(00)00047-6) 11185700

[17] Armen RS, Uitto OD, Feller SE. 1998 Phospholipid component volumes: determination and application to bilayer structure calculations. Biophys. J. **75**, 734–744. (10.1016/S0006-3495(98)77563-0) 9675175 PMC1299748

[18] Hughes AV, Ciesielski F, Kalli AC, Clifton LA, Charlton TR, Sansom MSP, Webster JRP. 2016 On the interpretation of reflectivity data from lipid bilayers in terms of molecular-dynamics models. Acta Crystallogr. Sect. D Struct. Biol. **72**, 1227–1240. (10.1107/s2059798316016235) 27917824

[19] McCluskey AR, Grant J, Smith AJ, Rawle JL, Barlow DJ, Lawrence MJ, Parker SC, Edler KJ. 2019 Assessing molecular simulation for the analysis of lipid monolayer reflectometry. J. Phys. Commun. **3**, 075001. (10.1088/2399-6528/ab12a9)

[20] Darré L, Iglesias-Fernandez J, Kohlmeyer A, Wacklin H, Domene C. 2015 Molecular dynamics simulations and neutron reflectivity as an effective approach to characterize biological membranes and related macromolecular assemblies. J. Chem. Theory Comput. **11**, 4875–4884. (10.1021/acs.jctc.5b00635) 26574275

[21] Mori T, Miyashita N, Im W, Feig M, Sugita Y. 2016 Molecular dynamics simulations of biological membranes and membrane proteins using enhanced conformational sampling algorithms. Biochim. Et Biophys. Acta **1858**, 1635–1651. (10.1016/j.bbamem.2015.12.032) PMC4877274 26766517

[22] Laio A, Parrinello M. 2002 Escaping free-energy minima. Proc. Natl Acad. Sci. USA **99**, 12562–12566. (10.1073/pnas.202427399) 12271136 PMC130499

[23] Hamelberg D, Mongan J, McCammon JA. 2004 Accelerated molecular dynamics: a promising and efficient simulation method for biomolecules. J. Chem. Phys. **120**, 11919–11929. (10.1063/1.1755656) 15268227

[24] Sugita Y, Okamoto Y. 1999 Replica-exchange molecular dynamics method for protein folding. Chem. Phys. Lett. **314**, 141–151. (10.1016/S0009-2614(99)01123-9)

[25] Costa MGS, Batista PR, Gomes A, Bastos LS, Louet M, Floquet N, Bisch PM, Perahia D. 2023 MDexciteR: enhanced sampling molecular dynamics by excited normal modes or principal components obtained from experiments. J. Chem. Theory Comput. **19**. (10.1021/acs.jctc.2c00599) 36622950

[26] Hénin J, Lelièvre T, Shirts MR, Valsson O, Delemotte L. 2022 Enhanced sampling methods for molecular dynamics simulations [Article v1.0]. LiveCoMS **4**, 1583. (10.33011/livecoms.4.1.1583)

[27] Marrink SJ, Risselada HJ, Yefimov S, Tieleman DP, de Vries AH. 2007 The MARTINI force field: coarse grained model for biomolecular simulations. J. Phys. Chem. B **111**, 7812–7824. (10.1021/jp071097f) 17569554

[28] Orsi M, Essex JW. 2011 The ELBA force field for coarse-grain modeling of lipid membranes. PloS One **6**, e28637. (10.1371/journal.pone.0028637) 22194874 PMC3241685

[29] Li X, Gao L, Fang W. 2016 Dissipative particle dynamics simulations for phospholipid membranes based on a four-to-one coarse-grained mapping scheme. PloS One **11**, e0154568. (10.1371/journal.pone.0154568) 27137463 PMC4854440

[30] Olsson S, Noé F. 2017 Mechanistic models of chemical exchange induced relaxation in protein NMR. J. Am. Chem. Soc. **139**, 200–210. (10.1021/jacs.6b09460) 27958728

[31] Childers MC, Daggett V. 2018 Validating molecular dynamics simulations against experimental observables in light of underlying conformational ensembles. J. Phys. Chem. B **122**, 6673–6689. (10.1021/acs.jpcb.8b02144) 29864281 PMC6420231

[32] Ranson NA, Farr GW, Roseman AM, Gowen B, Fenton WA, Horwich AL, Saibil HR. 2001 ATP-bound states of GroEL captured by cryo-electron microscopy. Cell **107**, 869–879. (10.1016/s0092-8674(01)00617-1) 11779463

[33] Cuello LG *et al*. 2010 Structural basis for the coupling between activation and inactivation gates in K+ channels. Nature **466**, 272–275. (10.1038/nature09136) 20613845 PMC3033755

[34] Birkner JP, Poolman B, Koçer A. 2012 Hydrophobic gating of mechanosensitive channel of large conductance evidenced by single-subunit resolution. Proc. Natl Acad. Sci. USA **109**, 12944–12949. (10.1073/pnas.1205270109) 22826215 PMC3420157

[35] Perkins SJ *et al*. 2016 Atomistic modelling of scattering data in the Collaborative Computational Project for Small Angle Scattering (CCP-SAS). J. Appl. Crystallogr. **49**, 1861–1875. (10.1107/S160057671601517X) 27980506 PMC5139988

[36] Hald Albertsen C, Kulkarni JA, Witzigmann D, Lind M, Petersson K, Simonsen JB. 2022 The role of lipid components in lipid nanoparticles for vaccines and gene therapy. Adv. Drug Deliv. Rev. **188**, 114416. (10.1016/j.addr.2022.114416) 35787388 PMC9250827

[37] Langeveld SAG, Schwieger C, Beekers I, Blaffert J, van Rooij T, Blume A, Kooiman K. 2020 Ligand distribution and lipid phase behavior in phospholipid-coated microbubbles and monolayers. Langmuir **36**, 3221–3233. (10.1021/acs.langmuir.9b03912) 32109064 PMC7279639

[38] Hollinshead CM, Harvey RD, Barlow DJ, Webster JRP, Hughes AV, Weston A, Lawrence MJ. 2009 Effects of surface pressure on the structure of distearoylphosphatidylcholine monolayers formed at the air/water interface. Langmuir **25**, 4070–4077. (10.1021/la8028319) 19714892

[39] Jo S, Kim T, Iyer VG, Im W. 2008 CHARMM‐GUI: A web‐based graphical user interface for CHARMM. J. Comput. Chem. **29**, 1859–1865. (10.1002/jcc.20945) 18351591

[40] Klauda JB, Venable RM, Freites JA, O’Connor JW, Tobias DJ, Mondragon-Ramirez C, Vorobyov I, MacKerell AD, Pastor RW. 2010 Update of the CHARMM all-atom additive force field for lipids: validation on six lipid types. J. Phys. Chem. B **114**, 7830–7843. (10.1021/jp101759q) 20496934 PMC2922408

[41] Jorgensen WL, Chandrasekhar J, Madura JD, Impey RW, Klein ML. 1983 Comparison of simple potential functions for simulating liquid water. J. Chem. Phys. **79**, 926–935. (10.1063/1.445869)

[42] Yu Y, Krämer A, Venable RM, Simmonett AC, MacKerell AD, Klauda JB, Pastor RW, Brooks BR. 2021 Semi-automated optimization of the CHARMM36 lipid force field to include explicit treatment of long-range dispersion. J. Chem. Theory Comput. **17**, 1562–1580. (10.1021/acs.jctc.0c01326) 33620214 PMC8059446

[43] Essmann U, Perera L, Berkowitz ML, Darden T, Lee H, Pedersen LG. 1995 A smooth particle mesh Ewald method. J. Chem. Phys. **103**, 8577–8593. (10.1063/1.470117)

[44] Phillips JC *et al*. 2005 Scalable molecular dynamics with NAMD. J. Comput. Chem. **26**, 1781–1802. (10.1002/jcc.20289) 16222654 PMC2486339

[45] de Souza RM, Romeu FC, Ribeiro MCC, Karttunen M, Dias LG. 2022 Osmotic method for calculating surface pressure of monolayers in molecular dynamics simulations. J. Chem. Theory Comput. **18**, 2042–2046. (10.1021/acs.jctc.2c00109) 35254819

[46] Prabhu J, Singh AP, Vanni S. 2023 An in silico osmotic pressure approach allows characterization of pressure–area isotherms of lipid monolayers at low molecular areas. Soft Matter **19**, 3377–3385. (10.1039/d2sm01419j) 37102755 PMC10170484

[47] Alberginia 2020 Made2Reflect. See https://gitlab.com/alberginia/Made2Reflect.

[48] Cousin F, Fadda G. 2020 An introduction to neutron reflectometry. EPJ Web Conf. (eds S Combet, G Schirò), **236**, 04001. (10.1051/epjconf/202023604001)

[49] Benattar JJ, Daillant J, Bosio L. 1991 Phase transitions, elasticity and capillary waves in Langmuir monolayers on water: an X-ray optical study. Phase Transitions **30**, 79–90. (10.1080/01411599108207966)

[50] See https://github.com/RascalSoftware/RAT.

[51] Shahmoradi A, Bagheri F. 2020 ParaDRAM: a cross-language toolbox for parallel high-performance delayed-rejection adaptive metropolis Markov chain Monte Carlo simulations. arXiv (10.48550/arXiv.2008.09589)

[52] Sivia DS, Webster JRP. 1998 The Bayesian approach to reflectivity data. Phys. B **248**, 327–337. (10.1016/s0921-4526(98)00259-2)

[53] Baoukina S, Monticelli L, Marrink SJ, Tieleman DP. 2007 Pressure−area isotherm of a lipid monolayer from molecular dynamics simulations. Langmuir **23**, 12617–12623. (10.1021/la702286h) 17973510

[54] Stachowicz-Kuśnierz A, Korchowiec B, Rogalska E, Korchowiec J. 2022 The lung surfactant activity probed with molecular dynamics simulations. Adv. Colloid Interface Sci. **304**, 102659. (10.1016/j.cis.2022.102659) 35421637

[55] Dabkowska AP, Talbot JP, Cavalcanti L, Webster JRP, Nelson A, Barlow DJ, Fragneto G, Lawrence MJ. 2013 Calcium mediated interaction of calf-thymus DNA with monolayers of distearoylphosphatidylcholine: a neutron and X-ray reflectivity study. Soft Matter **9**, 7095. (10.1039/c3sm50350j)

[56] Miyoshi T, Kato S. 2015 Detailed analysis of the surface area and elasticity in the saturated 1,2-diacylphosphatidylcholine/cholesterol binary monolayer system. Langmuir **31**, 9086–9096. (10.1021/acs.langmuir.5b01775) 26255826

[57] Duncan SL, Larson RG. 2008 Comparing experimental and simulated pressure-area isotherms for DPPC. Biophys. J. **94**, 2965–2986. (10.1529/biophysj.107.114215) 18199666 PMC2275714

[58] Kubo I, Adachi S, Maeda H, Seki A. 2001 Phosphatidylcholine monolayers observed with Brewster angle microscopy and π-A isotherms. Thin Solid Films **393**, 80–85. (10.1016/s0040-6090(01)01101-4)

[59] Jyoti A, Prokop RM, Li J, Vollhardt D, Kwok DY, Miller R, Möhwald H, Neumann AW. 1996 An investigation of the compression rate dependence on the surface pressure-surface area isotherm for a dipalmitoyl phosphatidylcholine monolayer at the air/water interface. Colloids Surfaces **116**, 173–180. (10.1016/0927-7757(96)03589-3)

[60] Klopfer KJ, Vanderlick TK. 1996 Isotherms of dipalmitoylphosphatidylcholine (DPPC) monolayers: features revealed and features obscured. J. Colloid Interface Sci. **182**, 220–229. (10.1006/jcis.1996.0454)

[61] Hardy NJ, Richardson TH, Grunfeld F. 2006 Minimising monolayer collapse on Langmuir troughs. Colloids Surfaces **284–285**, 202–206. (10.1016/j.colsurfa.2006.02.001)

[62] arwelHughes, & valerialosasso. 2024 valerialosasso/scripts_MD_NR: Scripts used to analyse MD and NR data. (Scripts_MD_NR). Zenodo. See 10.5281/zenodo.14068321.

[63] Losasso V. 2024 Simulations [Data set]. Zenodo. See 10.5281/zenodo.10810115.

